# Identification of novel RHPS4-derivative ligands with improved toxicological profiles and telomere-targeting activities

**DOI:** 10.1186/s13046-014-0081-x

**Published:** 2014-10-06

**Authors:** Angela Rizzo, Sara Iachettini, Pasquale Zizza, Chiara Cingolani, Manuela Porru, Simona Artuso, Malcolm Stevens, Marc Hummersone, Annamaria Biroccio, Erica Salvati, Carlo Leonetti

**Affiliations:** Experimental Chemotherapy Laboratory, Regina Elena National Cancer Institute, via delle Messi d’Oro 156, 00158 Rome, Italy; School of Pharmacy, University of Nottingham, Nottingham, NG7 2RD UK; Pharminox Ltd, Biocity, Pennyfoot St, Nottingham, NG1 1GF UK

**Keywords:** Telomere-targeting agents, G-quadruplex, Chemotherapy

## Abstract

The pentacyclic acridinium salt RHPS4 (3,11-difluoro-6,8,13-trimethyl-8*H*-quino [4,3,2-*kl*] acridinium methosulfate, compound **1**) is one of the most interesting DNA G-quadruplex binding molecules due to its high efficacy in tumor cell growth inhibition both in *in vitro* models and *in vivo* against human tumor xenografts in combination with conventional chemotherapeutics. Despite compound **1** having desirable chemical and pharmaceutical properties, its potential as a therapeutic agent is compromised by off-target effects on cardiovascular physiology. In this paper we report a new series of structurally-related compounds which were developed in an attempt to minimize its off-target profile, but maintaining the same favorable chemical and pharmacological features of the lead compound. By performing a comparative analysis it was possible to identify which derivatives had the following properties: (i) to show a reduced capacity in respect to compound **1** to inhibit the hERG tail current tested in a patch clamp assay and/or to interact with the human recombinant β2 receptor; (ii) to maintain both a good G4-binding affinity and cancer cell selectivity; and (iii) to trigger DNA damage with specific telomere uncapping. These studies allowed us to identify a novel G4-stabilizing molecule, compound **8**, being characterized by reduced off-target effects and potent telomere on-target properties compared to the prototypic compound **1**. Moreover, compound **8** shares with compound **1** the same molecular mode of action and an anti-tumour activity specifically restricted to replicating cells, as evident with its particularly efficient activity in combination therapy with a topoisomerase I inhibitor. In conclusion, we have identified a new pentacyclic derivative **8** having suitable properties to be the focus of further investigations as a clinical candidate for cancer therapy.

## Background

DNA has played an historic role as a molecular target for the development of some effective chemotherapeutics producing a significant improvement in the survival of patients. However, unfortunately, adverse side effects have limited their clinical potential. Consequently, much effort has been invested into finding novel agents that are more selective for cancer-specific DNA targets. Secondary DNA structures, such as G-quadruplex (G4), higher-order four-stranded structures, which can form in guanine-rich nucleic acid sequences, have recently emerged as a new class of molecular targets for developing DNA-interactive compounds as therapeutics in oncology and in other diseases [1]. Interest in the more general therapeutic significance of G4 has expanded during the past decade to include G4 structures not only at chromosome ends but also in the promoter sequences of a wide range of genes important in cell signalling, recognized as hallmarks of cancer. The broad concept of G4 DNA being therapeutically-susceptible hot-spots has recently been validated by their direct visualization in human cells [2] and by the finding that these structures can be stabilized in cells by small molecules [2,3].

As a result of research on telomeric G4 and the cellular consequence of targeting them with small molecules that stabilize these structures, their biological and therapeutic significance is well appreciated and continues to be an active field of drug discovery to identify appropriate modulators to be tested in patients. In this context, several chemotypes with different chemical structures have been developed showing good anti-tumor properties both *in vitro* and in xenografts [4,5]. However, notwithstanding the promising results obtained in preclinical models, the synthetic compound quarfloxin, CX-3543, is the sole G4-binding small molecule that has progressed to date to phase II clinical trial [6] and very recently Tetragene (www.tetragene.com) has in-licensed it for further clinical development.

Our pioneering studies have clearly reported that G4-interacting agents are more than simple telomerase inhibitors and that their direct target is rather the telomere *per se* than telomerase [7,8]. In particular, we have investigated thoroughly the antitumor properties and the molecular mechanism(s) of action of a G4 ligand, the pentacyclic acridine RHPS4 (3,11-difluoro-6,8,13-trimethyl-8*H*-quino [4,3,2-*kl*] acridinium methosulfate, compound **1**). We observed initially that, in addition to its telomerase-inhibitory properties, this drug exerts an anticancer effect by impairing telomere replication with consequent telomeric chromatin alteration leading to the activation of a strong DNA damage response at telomeres [7-9]. Compound **1** is also one of the most effective and selective G4 ligands, showing single agent antitumoral activity with a good toxicological profile in a variety of human tumor xenografts in mice, and able to potentiate the antitumoral efficacy of topoisomerase I inhibitors and, spectacularly so, in a triple combination with irinotecan and a PARP-1 inhibitor [10-14].

Recently, we identified a G-rich sequence within the proximal promoter region of vegfr-2, able to form an antiparallel G4 structure that can be efficiently stabilized by RHPS4 with the consequence reduction of VEGFR-2 expression, thus resulting in the impairment of the angiogenic process [15]. Notwithstanding the fact that compound **1** has been documented in preclinical studies as a promising G4 ligand having many of the attributes of an ideal pharmaceutical [16], this compound did not progress to clinical trials since our recent study demonstrated some undesirable off-target effects. In fact, experiments performed on guinea pig showed cardiotoxicity probably related to the interaction of compound **1** with the β2 adrenergic receptor and M1, M2 and M3 muscarinic receptors, together with a potent inhibition of the hERG (human Ether-a-go-go Related Gene) tail current [17]. Through careful structural modifications, two second-generation molecules with significantly improved off-target profiles were identified [17] giving hope that it may be possible to develop a new agent from this pentacyclic class with minimal off-target liabilities. In this paper we report that a new series of compounds with antitumor properties comparable to compound **1**, coupled with improved toxicological profiles, thus identifying new possible candidates for clinical application.

## Methods

### Compounds

Compounds **2**–**10** were obtained from Pharminox Ltd, Biocity, Pennyfoot St, Nottingham NG 1 1GF, UK. Details of synthetic methods have been published (International Patent Application No. PCT/GB2011/051845 and PCT/GB2012/051467).

### Biosensor-surface plasmon resonance (SPR) studies

Oligonucleotides 5’-biotin-d [AG_3_ (T_2_AG_3_)_3_] quadruplex and 5’-biotin-CGA_3_T_3_C(CT)_2_GA_3_T_3_CG were purchased from Midland Certified Reagent Company (Midland, TX). Purification of DNA, preparation of solutions, collection of data, and analysis of results were conducted according to methods adopted in an earlier study [18].

### Receptor inhibition

hEGR study and the M2 and the β2 receptor inhibition assay were performed as previously reported [17,19].

### Cells and culture conditions

Normal WI-38 diploid human lung fibroblasts and the human colorectal adenocarcinoma HT29 cells were obtained from American Type Culture Collection (Manassas, VA, USA). BJ fibroblasts expressing hTERT and SV40 early region (BJ-EHLT) were obtained as previously reported [8]. Cells were grown in Dulbecco Modified Eagle Medium (D-MEM, Invitrogen Carlsbad, CA, USA) supplemented with 10% fetal calf serum, 2 mM L-glutamin and antibiotics.

### Cell proliferation

MTT (3-(4,5-Dimethylthiazol-2-yl)-2,5-diphenyltetrazolium Bromide) assay was performed in treated and untreated cells for 96 hours. Cells were incubated with MTT solution (Sigma-Aldrich), and the purple formazan crystals were dissolved in isopropanol. Optical densities (OD) at 540 nm was determined on microplate reader.

### Cytotoxic assay

The HT29 cells were seeded in 60 mm- Petri dishes at a density of 5x10^4^ cells/ plate in DMEM medium plus 10% serum FCS. After 24 hours cells were exposed to the following drugs: Ethyl-10-hydroxy-camptothecin (SN-38; 0.2, 0.4, 0.8 μM for 2 hrs), compound **8** (at 0.1, 0.2 and 0.4 μM for 96 hours) and compound **1** (0.5 and 1 μM for 96 hours). In the combination experiments the two different sequence of drug administration were evaluated: campthotecin followed by G-quadruplex ligands and the inverse sequence at fixed equipotent ratios. The medium containing the first drug was removed and replaced with fresh medium containing the second drug. Colony forming ability was evaluated as previously reported [20].

### Immunofluorescence

Cells were fixed in 2% formaldehyde and permeabilized in 0.25% Triton X100 in PBS for 5 min at room temperature. For immunolabeling, cells were incubated with primary antibody, then washed in PBS and incubated with the secondary antibodies. The following primary antibodies were used: pAb and mAb anti-TRF1(Abcam Ltd.; Cambridge UK); mAb (Upstate, Lake Placid, NY) and pAb anti-γH2AX (Abcam); mAb anti-PCNA (Sigma Chemicals, Milano, Italy). The following secondary antibody were used: TRITC conjugated Goat anti Rabbit, FITC conjugated Goat anti Mouse (Jackson ImmunoResearch Europe Ltd., Suffolk, UK). Fluorescence signals were recorded by using a Leica DMIRE2 microscope equipped with a Leica DFC 350FX camera and elaborated by a Leica FW4000 deconvolution software (Leica, Solms, Germany). This system permits to focus single planes inside the cell generating 3D highresolution images. For quantitative analysis of γH2AX positivity, 200 cells on triplicate slices were scored. For TIFs analysis, in each nucleus a single plane was analyzed and at least 50 nuclei per sample were scored.

### Statistical analysis

Synergism, additivity, and antagonism were assessed by isobologram analysis as reported previously [21]. Combination index (CI) values <0.9, > 0.9 < 1.2, and >1.2 indicate synergism, additivity, and antagonism, respectively. The Student’s *t*-test (unpaired, two-tailed) was used for comparing statistical differences. Differences were considered statistically significant when P < 0.05.

## Results and discussion

In previous papers we have detailed the chemical and pharmacological properties of the telomere-targeted agent 3,11-difluoro-6,8,13-trimethyl-8*H*-quino [4,3,2-*kl*] acridinium methosulphate (RHPS4, compound **1**). Unfortunately, its desirable potential as a therapeutic agent is compromised by cardiovascular effects that could be ameliorated to some extent in other related structures containing the pentacyclic acridinium pharmacophore [17].

**Figure 1:**
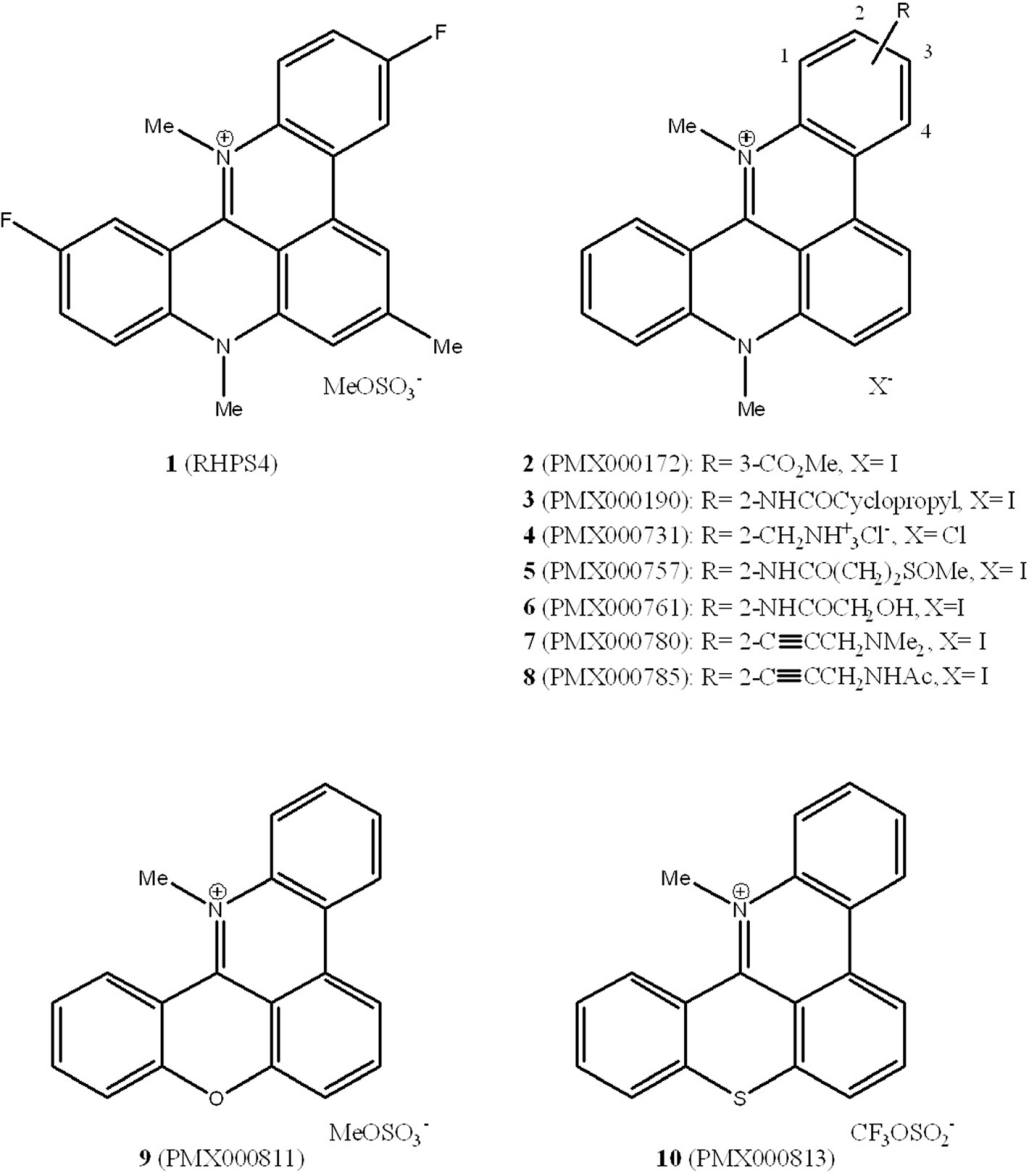
**Structures of 3,11-difluoro-6,8,13-trimethyl-8** ***H*** **-quino [4,3,2-** ***kl*** **] acridinium methosulfate (compound 1) and related chemotypes (2–10).**

In this paper we report preliminary results on further seven related structures (compounds **2–8**) with a range of different substituents at the 2- and 3-positions of the pentacyclic acridinium core (for numbering of the ring-system see Figure 1). In addition we include prototypic examples of two novel pentacycles (**9** and **10**) where the *N*-methyl fragment at position 8 in the pentacyclic acridinium system is replaced by an oxygen or sulphur atom, respectively. Full details of the synthetic routes to these novel structures (**2**–**10**) will be published elsewhere.

### On and off-target profiles of the new RHPS4-derivatives

The new G-quadruplex ligands were designed in an attempt to obtain molecules with improved toxicological and on-target profiles, but maintaining the same favorable chemical and pharmacological features of the lead compound **1**. With the aim of selecting the best variants, a step by step comparative analysis of their off- and on-target properties was performed. Initially the new molecules were tested for their ability to interact with and inhibit the human recombinant β2 adrenergic and M2 muscarinic receptors, and to reduce the hERG tail current in a patch clamp assay. As indicated in Table 1, the percents of inhibition were obtained by using each ligand at 1 and 10 μM respectively. Although all the compounds still maintained the capability of binding to the M2 receptor, most compounds, encouragingly, showed a reduced capacity to inhibiting the hERG tail current and/or interacting with the β2 receptor with respect to the lead molecule. Therefore, we might expect that the new ligands would impair cardiac functionality to a lesser extent than the lead molecule does.

**Table 1:** **On and off target profile of novel 1-derivative ligands**

**Compound ID**	**Off target receptor profile**			**DNA Affinity & Selectivity (Measured by SPR)**			**Cancer cell activity**	**“Normal” Cell activity**	
	**Muscarinic (M2) % inh @1** **μM**	**β2 adrenergic %** **inh @1** **μM**	**hERG % inh @10** **μM**	**Quadruplex DNA affinity Kx10** ^**6**^ **M** ^**−1**^	**Duplex DNA affinity Kx10** ^**6**^ **M** ^**−1**^	**Quadruplex/duplex ratio**	**HT-29 (GI**_**50**_ **/** **μm)**	**WI-38 (GI**_**50**_ **/** **μM)**	**Ratio PMX WI38 /HT29**
**1**	98	100	100	9.0	0.6	16.3	0.62	7.2	11,6
**2**	98	**11**	**41**	4.2	0,2	21	0.10	3.11	**31,1**
**3**	98,0	**13**	**74,0**	22,0	0,1	244,4	0,70	13,04	**18,5**
**4**	**65**	**1**	**15**	7.4	1,1	6,7	1,63	17.3	10,6
**5**	93	**10**	16	23	<0,1	>230	1,83	>30	ND
**6**	77	**8**	**35**	14,7	0,7	21	0,26	22,3	**85,7**
**7**	92	**0**	**37**	29,3	0,9	32,6	0,28	9.36	**33,4**
**8**	99	**2**	**40**	14,2	0,9	15,8	0,75	25,12	**33,5**
**9**	**24**	NE	86	1,5	<0,1	>15	0,21	5,11	**24,3**
**10**	49	NE	95	1,0	<0,1	>10	0,13	6,17	**47,5**

The relative binding affinity of each compound showed in Figure 1 for quadruplex and duplex DNA structures were measured by the Surface Plasmon Resonance (SPR). This technique takes advantage of the refractive index change elicits by the binding of the drug with the h-Tel quadruplex DNA sequence 5’-d[AGGG(TTAGGG)_3_]-3’ or an alternating hairpin duplex sequence immobilized on a sensor chip surface. As reported in Table 1, all the compounds analyzed, with the exception of compound **4**, showed a comparable or an enhanced quadruplex on duplex ratio relative to that of compound **1**, indicative of a global enhanced selectivity for G4 DNA structure.

### Biological characterization of new RHPS4-derivatives

These encouraging results in terms of potentially improved toxicological profile and DNA binding selectivity, encouraged us to conduct a more comprehensive biological characterization of the new ligands. Firstly, we investigated if they were able to efficiently promote growth inhibition of tumor cells without affecting the survival of normal cells *in vitro*. In Table 1 we report for each compound the concentration causing 50% of growth inhibition (GI_50_) calculated by performing a proliferation assay in the human colon cancer cell line HT-29 or in the human ‘normal’ lung WI-38 cells. The most efficacious agents in terms of normal/cancer cell selectivity were compounds **2**, **6**, **7** and **8**, all exhibiting (analogously to compound **1**) a low GI_50_ in HT-29 and an high GI_50_ in WI-38 treatment. On the basis of results described so far, we decided to exclude from the further analysis ligand **10** for its high value of hERG inhibition (95%) (Table 1).

**Figure 2:**
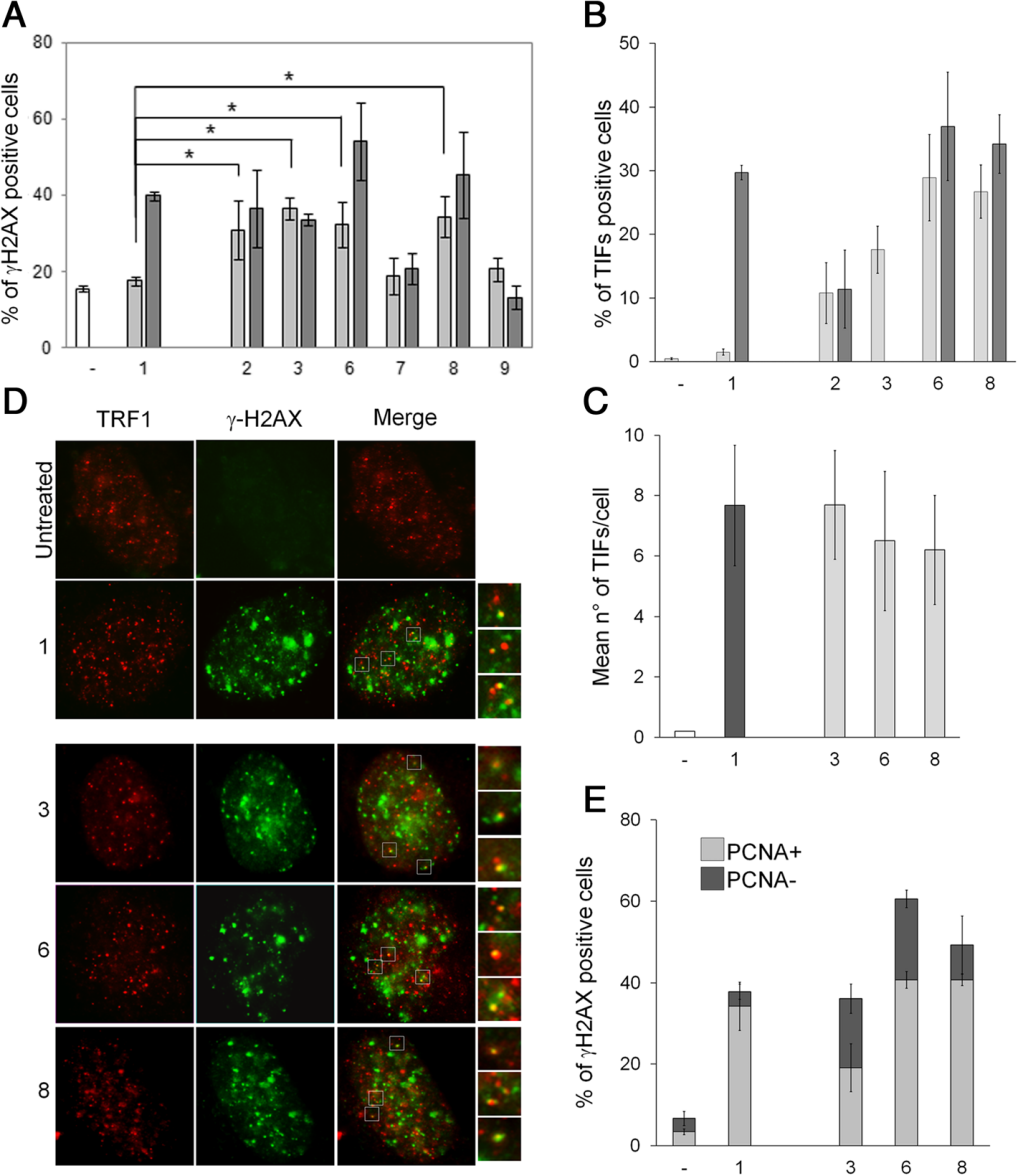
**DNA damage activation at telomeres.** BJ-EHLT fibroblasts were treated for 24 hrs with compound **1** and the indicated ligands at the doses 0.1 (light-grey bars) and 0.5 μM (dark-grey bars). Cells were processed for immunofluorescence (IF) using antibodies against γ-H2AX and TRF1 to mark DNA damage and telomeres respectively. Percentages of γ-H2AX- **(A)** and TIF-positive **(B)** treated and untreated cells are reported in the histograms. **(C)** Mean number of TIFs in the indicated samples. Cells with four or more γ-H2AX/TRF1 foci were scored as TIF positive. Error bars indicate the standard deviation. **(D)** Representative images of IF of untreated and 1, 3, 6 and 8-treated BJ-EHLT cells. Enlarged views of TIFs are reported on the right of the merged images. The images were acquired with a Leica Deconvolution microscope (magnification 100x). **(E)** Cells treated as in **(A)** were processed for IF of γ-H2AX and PCNA to mark replicating cells. Percentage of γ-H2AX+/PCNA- or γ-H2AX+/PCNA + nuclei in the indicated samples are reported in the histograms. The mean of three independent experiments with comparable results is shown.

In order to investigate the ability of the new G4-stabilizing agents to cause telomere uncapping, a two-step analysis was performed to establish if the compounds were able to induce DNA damage and, more significantly, if the DNA damage was localized to the telomeres. By performing immunofluorescence analysis on human transformed BJ fibroblasts (BJ-EHLT) to evaluate the phosphorylation of H2AX, a hallmark of DNA double strand break, we observed that all the compounds induced DNA damage at both 0,1 and 0,5 μM doses with the exception of compounds **7** and **9** (Figure 2A). Unfortunately, we were not able to test compounds **4**, **5** and the 0,5 μM dose of **3** as they were fluorescent in both red and green channels. The quantitative analysis revealed that the new ligands were more efficient in damaging DNA than compound **1**, since they elicited a significant percentage of cells positive for γ-H2AX at a dose of 0,1 μM (Figure 2A). To ascertain whether γ-H2AX was phosphorylated in response to dysfunctional telomeres, double immunofluorescence experiments were processed by confocal microscopy. We observed that some of the γ-H2AX foci induced by G4-ligands colocalized with TRF1, a good marker for interphase telomeres [18], forming the so-called telomere dysfunction-induced foci (TIFs) [19] (Figure 2B). Of note, quantitative analysis identified compounds **3**, **6** and **8** as the most potent in ability to specifically uncap telomeres: the percentage of cells with more than four γ-H2AX/TRF1 colocalizations reached, for compound **3,** about 18% in 0,1 μM treated cells and 40% in 0,5 μM (compounds **6** and **8**) (Figure 2B), with a mean of about seven TIFs per nucleus (Figure 2C). Intriguingly, even though the compounds **2**, **7** and **9** seemed to be promising in terms of off-target profile, G4-affinity and cancer cell selectivity, they did not cause significant telomere dysfunction (Figure 2B).

With the intent of further confirming if the new compounds resembled the same molecular mode of action of the lead chemotype **1** [9], we compared their capability to trigger a replication-dependent DNA damage − in particular, to determine which fraction of the cells formed γ-H2AX foci. We performed co-immunostaining to γ-H2AX and the proliferating cell nuclear antigen PCNA, which accumulates in the nucleus during S phase of cell cycle. In the case of compounds **1** and **8**, γ-H2AX foci formation was almost exclusively restricted to PCNA-positive, and so replicating, cells: in the case of the other drugs γ-H2AX foci formed both in PCNA-positive and -negative cells (Figure 2D), indicating that the compounds induced a cell cycle-independent DNA damage. At the end of our screening, we can conclude that most successful novel molecules in terms of telomere targeting as well as of improved toxicological profile compared to the original compound **1** were the ligands **6** and **8**. Of the new compounds, agent **8** showed a replication-dependent mode of action similar to compound **1**.

### Synergistc effect of compound 8 with a topoisomerase Inhibitor

**Figure 3:**
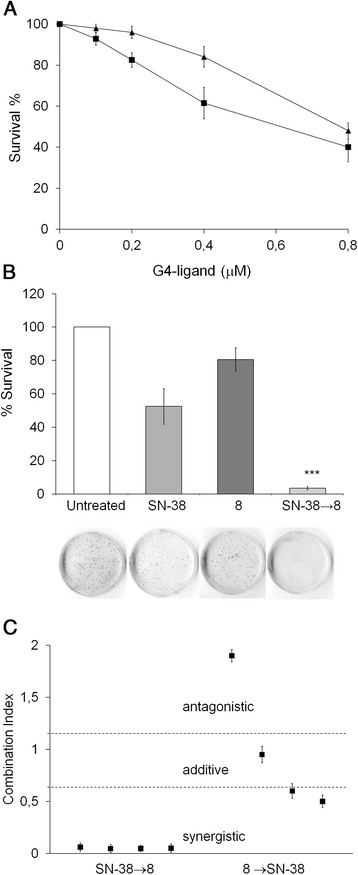
**Anti-tumor efficacy of compound 8 in single or combined administration with the topoisomerase I inhibitor SN-38. (A)** HT29 cells were exposed for 96 hrs to different doses (ranging from 0.1 to 0.8 μM) of the G4-ligand **1**(▲) or **8**(■). Surviving fractions were calculated as the ratio of absolute survival of the treated sample/absolute survival of the untreated sample. **(B)** HT29 cells were treated with 0.2 μM SN-38 for 2 hrs or with 0.2 μM 8 for 96 hours as single or in combined administration. In the histograms the surviving fractions calculated as in **(A)** are reported. Representative images of clonogenic ability of untreated or treated cells were showed below the histograms. **(C)** Combination Index for SN-38 and 8 was calculated by the Chou–Talalay method. Data plotted are CI at 50% (white squares), 75% (light gray squares), 90% (dark gray squares), and 95% (black squares) fraction killed. Data represent the means ± SD of 3 independent experiments.

We have previously reported that compound **1** in combination therapy with topoisomerase (TOPO) I inhibitors (Camptothecins) produced a synergistic antitumoral activitiy in *in vitro* and *in vivo* models [12,13]. Our published results support the hypothesis that this synergism relies on the role of TOPO I in relaxing the topological stress normally occurring during the progression of the replication fork and drastically increased at telomeres by the presence of G4 stabilizing agents. Tumor cells exposed to a TOPO I inhibitor prior to the administration of a G4 ligand were prevented or impaired in repairing dysfunctional telomeres, becoming more susceptible to cell death than if they received the single treatments, or the opposite sequence, of drug exposure. The study of *in vitro* interaction between agent **8** and ethyl-10-hydroxy-camptothecin (SN-38), the active metabolite of camptothecin Irinotecan, was preceded by experiments in which the cell colony-forming ability of the human colorectal adenocarcinoma HT29 cells was evaluated at different doses of compound **1** or **8**. Results reported in Figure 3A indicate that the new G4-ligand, at equal time of drug exposure (96 hours), inhibited cell survival in a dose-dependent manner like compound **1** but more efficiently so. Moreover, when HT29 cells were treated with different concentrations of SN-38 and compound **8**, a strong synergistic effect, with a Combination Index (CI) < 0.5, was observed when the first agent was followed by the G4-ligand, both at already the lowest dosages tested (Figure 3B-C and data not shown). As expected from the previously reported combination between compound **1** and SN-38, the inverse sequence of drug administration was less effective in reducing the tumor cell survival, eliciting only an additive or slight synergistic interaction (Figure 3C), thus further confirming the high mechanicistic analogy between compounds **1** and **8** when applied in combination therapy with a TOPO I inhibitor.

## Conclusions

In conclusion, the modifications of the prototype pentacyclic acridinium salt **1** allowed the synthesis and the selection of a novel promising G4-stabilizing telomere targeting agent (compound **8**), being superior to compound **1** both in toxicological profile and on-target properties, which could be a suitable compound for progression into clinical trials.
