# Erratum: Identification of novel RHPS4-derivative ligands with improved toxicological profiles and telomere-targeting activities

**DOI:** 10.1186/s13046-014-0101-x

**Published:** 2015-02-05

**Authors:** Angela Rizzo, Sara Iachettini, Pasquale Zizza, Chiara Cingolani, Manuela Porru, Simona Artuso, Malcolm Stevens, Marc Hummersone, Annamaria Biroccio, Erica Salvati, Carlo Leonetti

**Affiliations:** Experimental Chemotherapy Laboratory, Regina Elena National Cancer Institute, Nottingham, NG7 2RD, UK; School of Pharmacy, University of Nottingham, Nottingham, NG7 2RD, UK; Pharminox Ltd, Biocity, Pennyfoot St, Nottingham, NG1 1GF, UK

## Erratum

After the publication of this article [1] the authors noticed an error in the Acknowledgements section. The corrected Acknowledgments is included below. The authors apologise for any inconvenience.
